# The development and validation of a “5A” severity scale for predicting in-hospital mortality after accidental hypothermia from J-point registry data

**DOI:** 10.1186/s40560-019-0384-2

**Published:** 2019-05-03

**Authors:** Yohei Okada, Tasuku Matsuyama, Sachiko Morita, Naoki Ehara, Nobuhiro Miyamae, Takaaki Jo, Yasuyuki Sumida, Nobunaga Okada, Makoto Watanabe, Masahiro Nozawa, Ayumu Tsuruoka, Yoshihiro Fujimoto, Yoshiki Okumura, Tetsuhisa Kitamura, Shungo Yamamoto, Ryoji Iiduka, Kaoru Koike

**Affiliations:** 1 Department of Primary Care and Emergency Medicine, Graduate School of Medicine, Kyoto University, 606-8501, Yoshidakonoe-cho, Sakyo, Kyoto, Japan; 2 Department of Emergency and Critical Care Medicine, Japanese Red Cross Society, Kyoto Daini Hospital, Kyoto, Japan; 3 Department of Emergency Medicine, Kyoto Prefectural University of Medicine, Kyoto, Japan; 4 Senri Critical Care Medical Center, Saiseikai Senri Hospital, Suita, Japan; 5 Department of Emergency, Japanese Red Cross Society, Kyoto Daiichi Red Cross Hospital, Kyoto, Japan; 6 Department of Emergency Medicine, Rakuwa-kai Otowa Hospital, Kyoto, Japan; 7 Department of Emergency Medicine, Uji-Tokushukai Medical Center, Uji, Japan; 8 Department of Emergency Medicine, North Medical Center, Kyoto Prefectural University of Medicine, Kyoto, Japan; 9 Department of Emergency and Critical Care Medicine, National Hospital Organization, Kyoto Medical Center, Kyoto, Japan; 10 Department of Emergency and Critical Care Medicine, Saiseikai Shiga Hospital, Ritto, Japan; 11 Department of Emergency and Critical Care Medicine, Kyoto Min-Iren Chuo Hospital, Kyoto, Japan; 12 Department of Emergency Medicine, Yodogawa Christian Hospital, Osaka, Japan; 13 Department of Emergency Medicine, Fukuchiyama City Hospital, Fukuchiyama, Japan; 14 Division of Environmental Medicine and Population Sciences, Department of Social and Environmental Medicine, Graduate School of Medicine, Osaka University, Osaka, Japan; 15 Department of Healthcare Epidemiology, School of Public Health in the Graduate School of Medicine, Kyoto University, Kyoto, Japan

**Keywords:** Environmental emergency, Accidental hypothermia, Cardiac arrest

## Abstract

**Background:**

Accidental hypothermia is a serious condition that requires immediate and accurate assessment to determine severity and treatment. Currently, accidental hypothermia is evaluated using the Swiss grading system which uses core body temperature and clinical findings; however, research has shown that core body temperature is not associated with in-hospital mortality in urban settings. Therefore, we developed and validated a severity scale for predicting in-hospital mortality among urban Japanese patients with accidental hypothermia.

**Methods:**

Data for this multi-center retrospective cohort study were obtained from the J-point registry. We included patients with accidental hypothermia who were admitted to an emergency department. The total cohort was divided into a development cohort and validation cohort, based on the location of each institution. We developed a logistic regression model for predicting in-hospital mortality using the development cohort and assessed its internal validity using bootstrapping. The model was then subjected to external validation using the validation cohorts.

**Results:**

Among the 572 patients in the J-point registry, 532 were ultimately included and divided into the development cohort (*N* = 288, six hospitals, in-hospital mortality 22.0%) and the validation cohort (*N* = 244, six hospitals, in-hospital mortality 27.0%). The 5 “A” scoring system based on age, activities-of-daily-living status, near arrest, acidemia, and serum albumin level was developed based on the variables’ coefficients in the development cohort. In the validation cohort, the prediction performance was validated.

**Conclusion:**

Our “5A” severity scoring system could accurately predict the risk of in-hospital mortality among patients with accidental hypothermia.

**Electronic supplementary material:**

The online version of this article (10.1186/s40560-019-0384-2) contains supplementary material, which is available to authorized users.

## Background

Accidental hypothermia (AH) involves an unintentional decrease in core body temperature to ≤ 35 °C [1]. This condition is associated with high risks of hemodynamic collapse and mortality (24–40%) [2–4], as the cooling heart results in decreased cardiac output and electrical conduction abnormalities leading to life-threatening dysrhythmias, such as bradycardic atrial fibrillation or ventricular fibrillation [1]. Therefore, patients with AH must be immediately assessed to determine their severity and select appropriate advanced resuscitation and critical care techniques.

Although AH patients require immediate assessment of the severity and critical care, there is no established risk assessment tool specialized for AH patients. This might lead to inappropriate decision-making due to a lack of accurate information for the prognosis. The severity of AH is traditionally evaluated using the Swiss grading system [1] which is based on core body temperature and simple clinical findings. However, other research has indicated that core body temperature is not associated with in-hospital mortality in urban settings [2, 4, 5]. Moreover, mortality is known to be associated with various other factors, such as age, activities of daily living (ADL), hemodynamic instability, hyperkalemia, and acidemia [1, 2, 4–9]. Unfortunately, it is difficult to understand how these factors might influence mortality, especially in an emergency setting. Thus, a simple and user-friendly severity scale is needed to estimate mortality after AH in urban settings. The present study aimed to develop and validate a severity scaling system for predicting in-hospital mortality using data from Japanese patients who experienced AH in urban settings.

## Methods

This multi-center retrospective cohort study complied with the TRIPOD statement (Transparent Reporting of a Multivariable Prediction Model for Individual Prognosis or Diagnosis) regarding the reporting of the study’s methods and results [10].

### Data source

We obtained epidemiological and clinical information from the J-point registry database which collects data from a network of Japanese centers that treat patients with AH [2]. Eight centers are designated as Critical Care Medical Centers (CCMCs), and four sites are the emergency departments (EDs) of non-CCMC general hospitals in urban areas of the Kyoto, Osaka, and Shiga prefectures in Japan. Each year, the centers had a median of 19,651 ED visits (interquartile range 13,281–27,554 visits). In Japan, CCMCs are certified by the Ministry of Health, Labour and Welfare based on EDs that treat patients for shock, trauma, resuscitation, and critical care which serve approximately 500,000 residents in each region; in these CCMCs, advanced treatment like extracorporeal membrane oxygenation (ECMO) is generally available [11]. The non-CCMC centers are public or private general hospitals that cover a smaller regional community, and, generally, advanced treatment such as ECMO is unavailable.

The J-point registry includes patients who are retrospectively identified at each center using the International Classification of Diseases, Tenth Revision (ICD-10) code for hypothermia (T68). These patients were treated for hypothermia between April 1, 2011, and March 31, 2016, and had a body temperature of unknown or ≤ 35.0 °C. Patients were excluded from the registry if they or their family members explicitly refused to be included in the registry. Clinical data were extracted by emergency physicians using a predefined data extraction sheet. The collected data were re-checked by the J-Point Registry Working Group members and either confirmed or checked with the appropriate institution if there were concerns regarding the data’s validity. Based on these factors, 572 patients were registered in the J-point registry. The ethics committee of each center approved the registry protocol and retrospective analysis of de-identified data.

### Study population

The present study included adult patients (≥ 16 years old) with a core body temperature of ≤ 35 °C at ED admission and excluded patients with a non-AH core body temperature (> 35 °C or unknown) and missing data regarding age, sex, and mortality. The model was planned to undergo both internal and external validation [12, 13]. Thus, a development cohort was created based on centers from Kyoto city (four CCMCs and two non-CCMCs), while the validation cohort was created based on centers from Shiga and Osaka prefecture and Kyoto prefecture except for Kyoto city (four CCMCs and two non-CCMCs). This approach was selected because random sample splitting is not recommended for relatively small cohorts (to avoid over-fitting the data), which should instead be subdivided based on a time period or geographical location [12, 14]. The validation cohort was considered sufficient for external validation because the sample splitting was based on geographical location and not random allocation [12, 14].

### Data collection and patient outcomes

The institutions were categorized as CCMC or non-CCMC, and the annual number of ED visits, the average number of hospital beds, and patients’ characteristics including sex, age, independent or disturbed ADLs, and comorbidities were collected (Additional file 1). The patients’ clinical characteristics were defined as vital signs at hospital arrival (core body temperature, systolic blood pressure [SBP], and Glasgow [GCS] and Japan [JCS] Coma Scales) and biological data (serum pH, potassium [K^+^][mEq/L], and albumin [g/dL]). Details of the patients’ clinical characteristics are provided in Additional file 1. Treatment characteristics were defined as external and minimally invasive rewarming methods (warm intravenous fluid, forced warm air, warm blanket, and others) and active internal rewarming (lavage, intravascular rewarming device, and veno-venous and veno-arterial ECMO) (Additional file 1: Table S1). The outcome of interest was defined as in-hospital mortality, which was also determined retrospectively.

**Table 1:** Patient and institution characteristics

Parameters	Development cohort		Validation cohort		Total cohort	
	(*N* = 288)		(*N* = 244)		(*N* = 532)	
Male, *n* (%)	144	(50.0%)	126	(51.6%)	270	(50.8%)
Age, years, median (IQR)	79 (69–87)		79 (64–87)		79 (67–87)	
< 60, *n* (%)	37	(12.8%)	42	(19.3%)	79	(14.8%)
60–69	35	(12.2%)	37	(15.2%)	72	(13.5%)
70–79	76	(26.4%)	48	(19.7%)	124	(23.3%)
≥ 80	140	(48.6%)	117	(48.0%)	257	(48.3%)
Activities of daily living						
Independent *n* (%)	190	(66.0%)	178	(73.0%)	368	(69.2%)
Disturbance	96	(33.3%)	66	(27.0%)	162	(30.5%)
Missing	2	(0.7%)	0	(0.0%)	2	(0.4%)
Comorbidities						
Cardiovascular diseases, *n* (%)	126	(43.8%)	111	(45.5%)	237	(44.5%)
Neurological diseases	53	(18.4%)	40	(16.4%)	93	(17.5%)
Endocrine diseases	83	(28.8%)	47	(19.3%)	130	(24.4%)
Psychiatric diseases	55	(19.1%)	63	(25.8%)	118	(22.2%)
Malignant diseases	12	(4.2%)	4	(1.6%)	16	(3.0%)
Dementia	57	(19.8%)	51	(20.9%)	108	(20.3%)
Other	56	(19.4%)	38	(15.6%)	94	(17.7%)
External and minimally invasive rewarming						
Warm intravenous fluid, *n* (%)	223	(77.4%)	168	(68.9%)	391	(73.5%)
Forced warm air	80	(27.8%)	4	(1.6%)	84	(15.8%)
Warm environment, blanket	242	(84.0%)	222	(91.0%)	464	(87.2%)
Other	23	(8.0%)	15	(6.1%)	38	(7.1%)
Active internal rewarming						
Lavage, *n* (%)	29	(10.1%)	15	(6.1%)	44	(8.3%)
CHDF	4	(1.4%)	17	(7.0%)	21	(3.9%)
VV-ECMO	0	(0.0%)	2	(0.8%)	2	(0.4%)
VA-ECMO	3	(1.0%)	17	(7.0%)	20	(3.8%)
In-hospital mortality, *n* (%)	64	(22.2%)	66	(27.0%)	130	(24.4%)
Institution						
CCMC, *n* (%)	4	(66.7%)	4	(66.7%)	8	(66.7%)
ED visit, median (IQR)	19,651 (12,076–28,439)		20,798 (12,319–28,801)		19,651 (13,252–27,811)	
Number of beds, median (IQR)	640 (513–825)		374 (331–512)		510 (364–668)	

*IQR* interquartile range, *CHDF* continuous hemodiafiltration, *VV* veno-venous, *VA* veno-arterial, *ECMO* extracorporeal membrane oxygenation, *CCMC* critical care medical center, *ED Visit* annual number of emergency department visit

### Prognostic variable selection, data preparation, and handling missing data

Based on previous studies and expert opinions, we selected the admission values for age, ADL, body temperature, level of consciousness, hemodynamic state, serum pH, albumin, and K^+^ as potential predictor candidates of in-hospital mortality [1, 2, 4–9]. To ensure that the model is user-friendly, especially for emergency settings, we categorized the potential covariates based on their normal limit or commonly used ranges. Level of consciousness was classified as mild (GCS 13–15 or JCS 0–3), moderate (GCS 9–12 or JCS 10–30), and severe (GCS < 9 or JCS 100–300). Details of the JCS are described in Additional file 1. A status of “near arrest” was defined as an SBP of ≤ 60 mmHg, unmeasurable values, and cardiac arrest. In terms of missing values, variables with < 3% missing data were analyzed based on complete case analysis as such an analysis might then be feasible [15]. If missing values were > 3%, missing data were categorized as “unknown,” because unmeasured values might be informative in clinical settings (e.g., in minor cases, blood gas analysis tends to be omitted). Tables 1 and 2 show the distributions of the covariate categories for each cohort.

**Table 2:** Vital sign and laboratory data on admission

Parameters	Development cohort		Validation cohort		Total cohort	
	(*N* = 288)		(*N* = 244)		(*N* = 532)	
Systolic blood pressure (mm Hg)						
≧ 90, *n* (%)	212	(73.6%)	156	(63.9%)	368	(69.2%)
61–90	45	(15.6%)	45	(18.4%)	90	(16.9%)
Near cardiac arrest	31	(10.8 %)	43	(17.6%)	74	(13.9%)
Body temperature (°C), median (IQR)	30.7 (28.3–32.6)		31.0 (28–32.7)		30.8 (28.1–32.6)	
35–32, *n* (%)	98	(34.0%)	77	(31.6%)	175	(32.9%)
32–28	124	(43.1%)	104	(42.6%)	228	(42.9%)
< 28	66	(22.9%)	63	(25.8%)	129	(24.2%)
Disturbance of consciousness						
Mild, *n* (%)	110	(38.2%)	100	(41.0%)	210	(39.5%)
Moderate	90	(31.3%)	70	(28.7%)	160	(30.1%)
Severe	85	(29.5%)	68	(27.9%)	153	(28.8%)
Missing	3	(1.0%)	6	(2.5%)	9	(1.7%)
pH, median (IQR)	7.31 (7.25–7.37)		7.31 (7.21–7.37)		7.31 (7.23–7.37)	
> 7.35, *n* (%)	85	(29.5%)	69	(28.3%)	154	(28.9%)
7.20–7.35	118	(41.0%)	98	(40.2%)	216	(40.6%)
< 7.20	42	(14.6%)	53	(21.7%)	95	(17.9%)
Missing	43	(14.9%)	24	(9.8%)	67	(12.6%)
K^+^ (mmol/l), median (IQR)	4.2 (3.6–4.7)		4.0 (3.5–4.6)		4.1 (3.6–4.7)	
< 3.5, *n* (%)	59	(20.5%)	54	(22.1%)	113	(21.2%)
3.5–5.5	198	(68.8%)	155	(63.5%)	353	(66.4%)
> 5.5	30	(10.4%)	28	(11.5%)	58	(10.9%)
Missing	1	(0.3%)	7	(2.9%)	8	(1.5%)
Albumin (g/dl), median (IQR)	3.4 (2.9–4.0)		3.5 (2.9–4.0)		3.4 (2.9–4.0)	
> 3	163	(56.6%)	142	(58.2%)	305	(57.3%)
≤ 3	77	(26.7%)	61	(25.0%)	138	(25.9%)
Missing	48	(16.7%)	41	(16.8%)	89	(16.7%)

In disturbance of consciousness, mild: Glasgow coma scale (GCS) 13–15, or Japan coma scale (JCS) 0–3, moderate: GCS 9–12, or JCS 10–30, severe: GCS < 9, or JCS 100–300

*IQR* interquartile range, near arrest: systolic blood pressure ≤ 60 mmHg, unmeasurable, or cardiac arrest

We did not calculate the required sample size, because the J-point registry contains the largest number of AH cases among the available literature, and we aimed to empirically include all available data to maximize the model’s power and generalizability [14]. There is a consensus on the importance of having an adequate sample size; however, there is no generally accepted approach for estimating the required sample size when developing and validating risk prediction models [14].

### Development and evaluation of the prediction model

In the development cohort, predictors were selected using a stepwise backward method based on the lowest Akaike’s information criterion from the potential predictor candidates mentioned above. It allowed us to develop a parsimonious predictor model for variable retention, and multivariable logistic regression was subsequently applied. Backward elimination is generally preferred as an automated selection procedure because all correlations between the predictors are considered in the modeling procedure [14]. Each variable’s coefficient *β* and odds ratio were reported with the 95% confidence interval (CI). The model’s performance was evaluated based on Somers’ D_xy_, the C index, the *R*^2^ value, the calibration intercept and slope, and the Brier score. Calibration plots were also created to graphically depict the association between the predicted and observed in-hospital mortality rates based on locally weighted scatterplot smoothing [13]. Internal validation involved a bootstrapping procedure using 200 samples drawn with replacement from the original sample [13].

The fixed model was applied to the validation cohort for external validation, and the discrimination and calibration performances were compared to those from the development cohort. Finally, we set the clinically useful simplified risk stratification using a simple integer risk score based on each variable’s coefficient *β* [13]. To assess discrimination performance, we compared the c-index of our risk scoring system with that of the core body temperature on admission, which is categorized by the Swiss grading system, commonly used to assess the severity in AH [1]. The diagnostic abilities [sensitivity, specificity, positive likelihood ratio (LR+) and negative likelihood ratio (LR−)] of each score were calculated. The calibration performance of risk stratification was graphically evaluated in terms of the relationship between the predicted and observed in-hospital mortality. All statistical results were considered significant at two-sided *P* values of < 0.05. Statistical analyses were performed using JMP Pro® 14 software (SAS Institute Inc., Cary, NC) and R software (version 1.1.456; R Studio Inc.) with the “rms” package [15].

## Results

### Patient characteristics

**Fig. 1:**
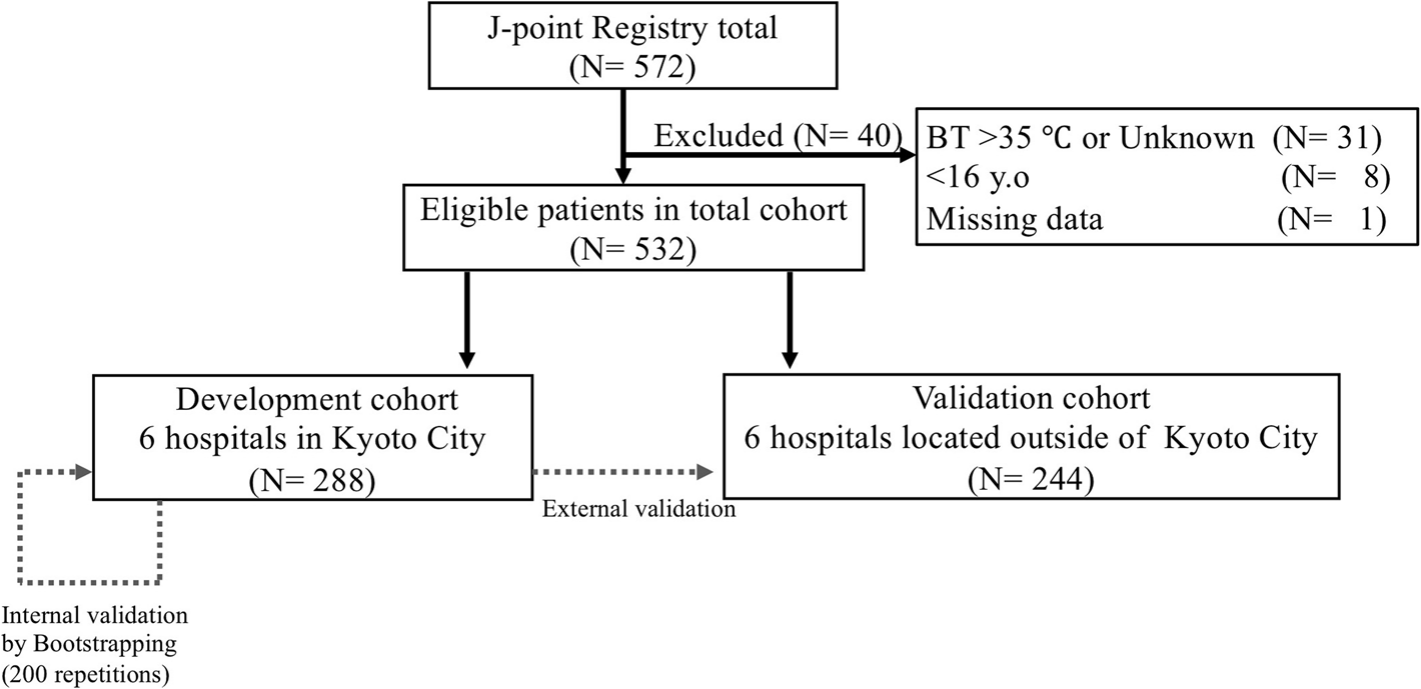
Study flow chart

Among the 572 patients in the J-point registry, we excluded 31 patients with a non-AH body temperature (> 35 °C or unknown), 8 non-adult patients (< 16 years old), and 1 patient with missing data. Thus, 532 patients were ultimately included, with an overall in-hospital mortality of 24.4%. The patients were then divided into the development cohort (*N* = 288, six hospitals [four CCMCs and two non-CCMCs], in-hospital mortality 22.0%) and the validation cohort (*N* = 244, six hospitals [four CCMCs and two non-CCMCs], in-hospital mortality 27.0%) (Fig. 1). The characteristics of the institutions and patients are shown in Tables 1 and 2, with the characteristics and distributions being generally similar between the cohorts. Missing values in pH and albumin were > 3% in each variable; thus, these missing values were categorized as “unknown,” and we conducted a complete case analysis.

### Performance and internal and external validation of the model

The 5 “A” predictors (age, ADL, near arrest state, acidemia, and albumin) were selected. The variables’ coefficient *β*, adjusted odds ratio with 95% CI, and the formula for predicted in-hospital mortality are shown in the Additional file 1: Table S2 and Formula. Evaluation of the model and the calibration plot in the development and validation cohorts were shown in Additional file 1: Table S3 and Figure S1 respectively, in Additional file 1. The calibration plot in both cohorts revealed a relatively good calibration, although the bias-corrected line revealed slight overestimation of the mortality risk.

### Risk scores and their performance

**Fig. 2:**
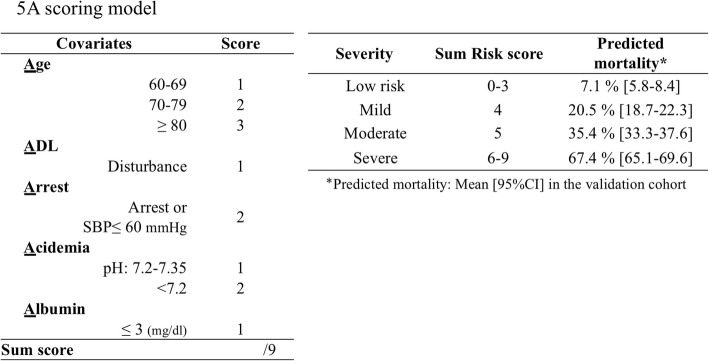
Calibration plot for each cohort. In the development cohort, the ideal dashed line reflects perfect calibration between the predicted and observed mortality. The apparent performance, indicated by the dotted line, reflects the calibrated performance of the model. The solid line reflects the bias-corrected performance based on bootstrapping. The validation cohort also has ideal dashed lines. The solid lines reflect the fitted logistic calibration curve. The dotted lines reflect a smooth nonparametric fit using a locally weighted scatter plot for smoothing

**Fig. 3:**
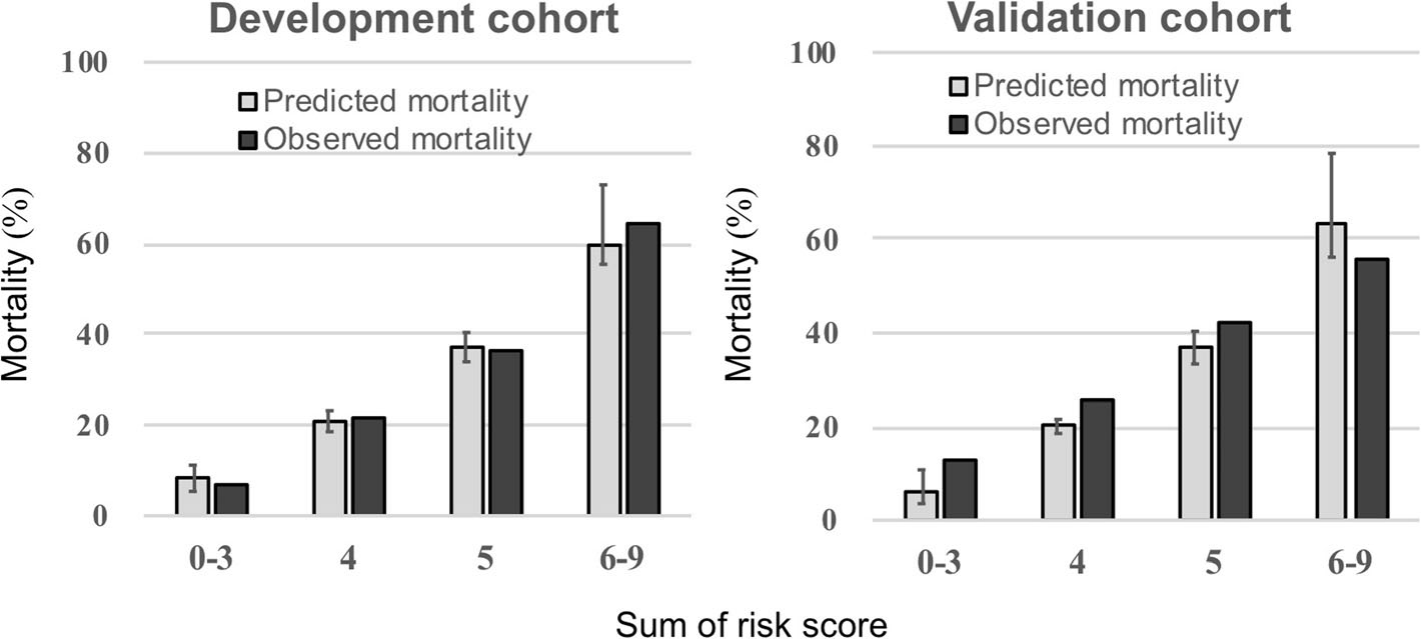
Predicted and observed mortality based on the 5A scoring system. The median predicted mortality rate is shown for the quartile-based sums of the risk scores in each cohort. The observed mortality rate reflected the proportion of in-hospital mortality. The predictions were well calibrated with the observations. The 5A scoring system provided a simple and rapid prediction of post-accidental hypothermia prognosis. ADL activities of daily living, SBP systolic blood pressure. Arrest was defined as SBP of ≤ 60 mmHg, unmeasurable values, and confirmed arrest

Based on the coefficient *β* of each predictor, a severity scoring scale was created (Fig. 2, Additional file 1: Table S4). The scoring system was based on age (60–69 years, + 1 point; 70–79 years, + 2 points; ≥ 80 years, + 3 points), ADL status (disturbed, + 1 point), hemodynamic status (near arrest, + 2 points), pH (7.35–7.2, + 1 point; < 7.2, + 2 points), and serum albumin level (≤ 3 mg/dL, + 1 point). The c-index of our scoring system was 0.776 and 0.731 in the development and validation cohorts, respectively. It was higher than that of the Swiss grading system (0.731 vs 0.558) in the validation cohort with statistical significance (Additional file 1: Table S5). The severity scale for predicting in-hospital mortality was defined as low risk (≤ 3 points), mild risk (4 points), moderate risk (5 points), and severe risk (≥ 6 points) (Fig. 2). In the validation cohort, the mean predicted mortality and observed mortality in each group were 7.1% (95% CI, 5.8–8.4%) and 12.6%, respectively, in the low-risk group; 20.5% (95% CI, 18.7–22.3%) and 26.3%, respectively, in the mild-risk group; 35.4% (95% CI, 33.3–37.6%) and 42.5%, respectively, in the moderate-risk group; and 67.4% (95% CI, 65.1–69.6%) and 55.6%, respectively, in the severe-risk group (Figs. 2 and 3). The diagnostic abilities of in-hospital mortality prediction in the low-risk group (≤ 3 points) were sensitivity 0.89 (95% CI, 0.82–0.97) and LR − 0.33 (95% CI, 0.16–0.68), which were suitable for rule-out, and in the severe-risk group (≥ 6 points), were specificity 0.91 (95% CI, 0.87–0.95) and LR + 3.37 (95% CI, 1.86–6.10), which were slightly suitable for rule-in (Table 3). Graphical evaluation of the severity scoring system revealed good calibration with the actual results in the validation cohort and was the same as in the development cohort (Fig. 3).

**Table 3:** Diagnostic ability of “5A” model for in-hospital mortality in validation cohort

Cutoff	Specificity	95%CI	Sensitivity	95%CI	LR+	95%CI	LR−	95%CI
8	0.99	(0.97–1.00)	0.09	(0.02–0.16)	NA	NA	NA	NA
7	0.98	(0.96–1.00)	0.21	(0.11–0.31)	9.44	(3.22–27.65)	0.81	(0.71–0.92)
6	0.91	(0.87–0.95)	0.30	(0.19–0.41)	3.37	(1.86–6.10)	0.77	(0.65–0.90)
5	0.78	(0.72–0.84)	0.56	(0.44–0.68)	2.56	(1.80–3.63)	0.56	(0.42–0.75)
4	0.54	(0.47–0.62)	0.79	(0.69–0.89)	1.73	(1.41–2.12)	0.39	(0.24–0.63)
3	0.33	(0.26–0.39)	0.89	(0.82–0.97)	1.33	(1.16–1.51)	0.33	(0.16–0.68)
2	0.16	(0.10–0.21)	0.97	(0.93–1.0)	1.15	(1.07–1.24)	0.19	(0.05–0.79)
1	0.06	(0.02–0.09)	0.98	(0.96–1.0)	NA	NA	NA	NA

*Sp* specificity, *Se* sensitivity*, LR+* positive likelihood ratio, *LR−* negative likelihood ratio, *CI* confidence interval

## Discussion

The present study revealed that a “5A” severity scoring scale (based on age, ADL, near arrest, acidemia, and albumin) had better ability to predict mortality after AH than the Swiss staging system based on the core body temperature, with good discrimination and calibration values based on internal and external validation. Furthermore, the severity scoring system will help emergency physicians to rapidly predict a patient’s prognosis and make management decisions. To the best of our knowledge, this is the first scale to be subjected to internal and external validation for predicting prognosis among patients with AH in urban areas.

### Previous literature and the present study’s strengths

Two reports have described methods for predicting prognosis after cardiac arrest due to AH [16, 17]. The ICE survival score (based on sex, asphyxiation, and serum K^+^) and the HOPE score (based on sex, asphyxia, age, K^+^, duration of cardiopulmonary resuscitation, and temperature) could predict prognosis after treatment using extracorporeal life support for AH cardiac arrest. However, these scores were developed based on a literature review, which included observational cohorts and case reports, and might have been affected by publication bias and selection bias. Moreover, these scores were not evaluated using bootstrapping as internal validation or a separate dataset for external validation which might have increased the risk of overfitting; thus, it raises questions regarding the applicability of these scores to other populations.

In contrast, the present study has several strengths. First, to our knowledge, ours is the largest cohort of patients with AH which allowed us to create two cohorts based on geographical location and subject the model to external validation. Second, we performed a bootstrapping procedure to assess overfitting and over-optimism during internal validation. Third, most patients were elderly which agrees with a recent report that indicated that most AH cases in Japan involve elderly people [2, 3, 9]. Population aging is a common public health issue in industrialized countries all over the world, and it is assumed that most victims of AH in developed countries will also be elderly. Most previous studies regarding AH have focused on younger patients [6–8, 16–19], with average ages of 35 years in the ICE score study and 36 years in the HOPE score study [16, 17]; therefore, these scores are not applicable for the general population. Thus, we believe that our model is more generalizable for patients who experience AH in urban areas.

### Interpretation

The present study evaluated clinically relevant variables that can be summarized as the “5A”s (age, ADL, near arrest, acidemia, and albumin). In this context, the patient’s values for age, ADL, and serum albumin may reflect a vulnerable physiological status, and these variables are commonly used as prognostic factors in critical care [20–23]. Hemodynamic instability and pH are also important factors in major critical care severity scoring scales [24] as they reflect the extent of vital organ hypoperfusion. Thus, we believe that the variables in our model could reflect hypothermia-related physiological changes. Similar to other studies [4], we did not incorporate body temperature as a predictor, as we hypothesized that a hypothermia-related decrease in organs’ oxygen consumption could protect them despite the presence of hypoperfusion, which would prevent body temperature from being strongly associated with prognosis.

During the model’s development, we used bootstrapping to account for slight over-optimism (e.g., correcting the C statistic from 0.794 to 0.746). We also found overestimation among the severe population in the bias-corrected calibration plot which appears to be mainly related to the small number of severe cases. The validation process also revealed slight overestimation among the severe population. Thus, we should interpret the findings carefully among severe cases.

### Clinical implications

We believe that this severity scoring scale allows clinicians to rapidly assess the severity of AH patients, provide patients and their families with accurate information, and improve their prognosis by more appropriately selecting severe cases which require advanced resuscitation and critical care. In outdoor activity (e.g., skiing, climbing) associated settings, most AH patients are healthy young athletes. In such situations, even if the probability of death is over 60%, aggressive treatment by physicians is reasonable. On the other hand, in urban areas, most AH patients are elderly [3, 9]. Since there is no established risk assessment tool available for their treatment, we are apprehensive about the appropriate treatment, because enough information for the prognosis is not available. For instance, elderly patients with impending death might be treated too invasively, without discussing the prognosis with their relatives, or those with good survival prospects might undergo early withdrawal of the treatment. The severity scoring system based on easily accessible data (“5A”) enables easy prognosis assessment by physicians. Aggressive treatment might be reasonable for patients found to be in the low-risk group (≤ 3 points), even if they are elderly. Physicians can easily identify the condition requiring critical care for those in the severe-risk group (≥ 6 points), and based on the possibility of prognosis, they can decide the indication after discussing with the patient’s relatives. Therefore, our risk scoring system can lead to rational decision-making based on the probability of prognosis evidence.

### Limitations

This study has several limitations. First, despite the generalizability of our model to similar urban areas, it is unclear if this model can be applied to other settings, for instance, an outdoor activity (i.e., skiing, mountain climbing, etc.) associated setting, in which most patients are healthy young athletes. Since our model was developed from an urban population, in which treatment is focused on elderly people who stay indoors [2, 9], the population and characteristics between these settings are totally different. A second limitation is the relatively small sample size which could have increased the risk of overfitting and optimism [14]. A third limitation is the absence of complete detailed data in the J-point registry regarding the AH event, the clinical course after admission, treatment, the neurological outcome, and the cause of death. Fourth, we did not compare the usefulness or diagnostic ability with general risk assessment tool for critically ill patients such as SOFA or APACHE2. Therefore, further research is needed to determine the validity, generalizability, and clinical usefulness of our model in other cohorts and to evaluate its clinical utility.

## Conclusion

The present study revealed that the 5A severity scale had good discrimination and calibration for predicting in-hospital mortality after AH based on internal and external validation. We believe that this severity scoring scale can be useful to rapidly assess the severity of patients with AH.

## Additional file

Additional file 1: The definition of patient characteristics and laboratory data. **Table S1.** The range of the laboratory data on arrival at the emergency department. **Table S2.** Coefficient β and adjusted odds ratio with 95% confidence intervals. **Table S3.** Model performance in the development cohort assessed by bootstrap and that in validation cohort. **Figure S1.** Calibration Plot in each cohort. **Table S4.** The conversion of the coefficient values to the score. **Table S5.** Comparing the discrimination performance in validation cohort. (DOCX 353 kb)

## References

[1] Brown DJ, Brugger H, Boyd J, Paal P (2012). Accidental hypothermia. N Engl J Med.

[2] Matsuyama T, Morita S, Ehara N, Miyamae N, Okada Y, Jo T, Sumida Y, Okada N, Watanabe M, Nozawa M, et al. Characteristics and outcomes of accidental hypothermia in Japan: the J-point registry. Emerg Med J. 2018. 10.1136/emermed-2017-207238 29886414

[3] Medicine. JAfA (2013). The clinical characteristics of hypothermic patients in the winter of Japan—the final report of hypothermia STUDY 2011. Journal of Japanese Association for Acute Medicine.

[4] Vassal T, Benoit-Gonin B, Carrat F, Guidet B, Maury E, Offenstadt G (2001). Severe accidental hypothermia treated in an ICU: prognosis and outcome. Chest.

[5] Okada Y, Matsuyama T, Morita S, Ehara N, Miyamae N, Jo T, Sumida Y, Okada N, Kitamura T, Iiduka R. Prognostic factors for patients with accidental hypothermia: a multi-institutional retrospective cohort study. Am J Emerg Med. 2018. 10.1016/j.ajem.2018.06.025 29950275

[6] Schaller MD, Fischer AP, Perret CH (1990). Hyperkalemia. A prognostic factor during acute severe hypothermia. Jama.

[7] Mair P, Kornberger E, Furtwaengler W, Balogh D, Antretter H (1994). Prognostic markers in patients with severe accidental hypothermia and cardiocirculatory arrest. Resuscitation.

[8] Silfvast T, Pettila V (2003). Outcome from severe accidental hypothermia in southern Finland--a 10-year review. Resuscitation.

[9] Morita S, Matsuyama T, Ehara N, Miyamae N, Okada Y, Jo T, Sumida Y, Okada N, Watanabe M, Nozawa M, et al. Prevalence and outcomes of accidental hypothermia among elderly patients in Japan: data from the J-point registry. Geriatr Gerontol Int. 2018. 10.1111/ggi.13502 30094918

[10] Collins GS, Reitsma JB, Altman DG, Moons KG (2015). Transparent reporting of a multivariable prediction model for individual prognosis or diagnosis (TRIPOD): the TRIPOD statement. Bmj.

[11] Ministry of Health, Labour and Welfare website [https://www.mhlw.go.jp/index.html].

[12] Steyerberg Ewout W., Harrell Frank E. (2016). Prediction models need appropriate internal, internal–external, and external validation. Journal of Clinical Epidemiology.

[13] Steyerberg EW (2009). Clinical prediction models: a practical approach to development, validation, and updating, vol.: hardcover. New York.

[14] Moons KG, Altman DG, Reitsma JB, Ioannidis JP, Macaskill P, Steyerberg EW, Vickers AJ, Ransohoff DF, Collins GS (2015). Transparent reporting of a multivariable prediction model for individual prognosis or diagnosis (TRIPOD): explanation and elaboration. Ann Intern Med.

[15] Harrell JFE: Regression modeling strategies: with applications to linear models, logistic regression, and survival analysis, vol.: [pbk.]. New York: Springer; 2010.

[16] Saczkowski RS, Brown DJA, Abu-Laban RB, Fradet G, Schulze CJ, Kuzak ND (2018). Prediction and risk stratification of survival in accidental hypothermia requiring extracorporeal life support: an individual patient data meta-analysis. Resuscitation.

[17] Pasquier M, Hugli O, Paal P, Darocha T, Blancher M, Husby P, Silfvast T, Carron PN, Rousson V (2018). Hypothermia outcome prediction after extracorporeal life support for hypothermic cardiac arrest patients: the HOPE score. Resuscitation.

[18] Farstad M, Andersen KS, Koller ME, Grong K, Segadal L, Husby P (2001). Rewarming from accidental hypothermia by extracorporeal circulation. A retrospective study. Eur J Cardiothorac Surg. Volume 20, edn. Germany.

[19] Ruttmann E, Weissenbacher A, Ulmer H, Muller L, Hofer D, Kilo J, Rabl W, Schwarz B, Laufer G, Antretter H (2007). Prolonged extracorporeal membrane oxygenation-assisted support provides improved survival in hypothermic patients with cardiocirculatory arrest. J Thorac Cardiovasc Surg.

[20] Heyland D, Cook D, Bagshaw SM, Garland A, Stelfox HT, Mehta S, Dodek P, Kutsogiannis J, Burns K, Muscedere J (2015). The very elderly admitted to ICU: a quality finish?. Crit Care Med.

[21] Yap FH, Joynt GM, Buckley TA, Wong EL (2002). Association of serum albumin concentration and mortality risk in critically ill patients. Anaesth Intensive Care.

[22] Sung J, Bochicchio GV, Joshi M, Bochicchio K, Costas A, Tracy K, Scalea TM (2004). Admission serum albumin is predicitve of outcome in critically ill trauma patients. Am Surg.

[23] Kim SW, Han HS, Jung HW, Kim KI, Hwang DW, Kang SB, Kim CH (2014). Multidimensional frailty score for the prediction of postoperative mortality risk. JAMA Surg.

[24] Knaus WA, Draper EA, Wagner DP, Zimmerman JE (1985). APACHE II: a severity of disease classification system. Crit Care Med.

