# Correction to: The development and validation of a “5A” severity scale for predicting in-hospital mortality after accidental hypothermia from J-point registry data

**DOI:** 10.1186/s40560-019-0388-y

**Published:** 2019-06-11

**Authors:** Yohei Okada, Tasuku Matsuyama, Sachiko Morita, Naoki Ehara, Nobuhiro Miyamae, Takaaki Jo, Yasuyuki Sumida, Nobunaga Okada, Makoto Watanabe, Masahiro Nozawa, Ayumu Tsuruoka, Yoshihiro Fujimoto, Yoshiki Okumura, Tetsuhisa Kitamura, Shungo Yamamoto, Ryoji Iiduka, Kaoru Koike

**Affiliations:** 1 Department of Primary Care and Emergency Medicine, Graduate School of Medicine, Kyoto University, 606-8501, Yoshidakonoe-cho, Sakyo, Kyoto, Japan; 2 Department of Emergency and Critical Care Medicine, Japanese Red Cross Society, Kyoto Daini Hospital, Kyoto, Japan; 3 Department of Emergency Medicine, Kyoto Prefectural University of Medicine, Kyoto, Japan; 4 Senri Critical Care Medical Center, Saiseikai Senri Hospital, Suita, Japan; 5 Department of Emergency, Japanese Red Cross Society, Kyoto Daiichi Red Cross Hospital, Kyoto, Japan; 6 Department of Emergency Medicine, Rakuwa-kai Otowa Hospital, Kyoto, Japan; 7 Department of Emergency Medicine, Uji-Tokushukai Medical Center, Uji, Japan; 8 Department of Emergency Medicine, North Medical Center, Kyoto Prefectural University of Medicine, Kyoto, Japan; 9 Department of Emergency and Critical Care Medicine, National Hospital Organization, Kyoto Medical Center, Kyoto, Japan; 10 Department of Emergency and Critical Care Medicine, Saiseikai Shiga Hospital, Ritto, Japan; 11 Department of Emergency and Critical Care Medicine, Kyoto Min-Iren Chuo Hospital, Kyoto, Japan; 12 Department of Emergency Medicine, Yodogawa Christian Hospital, Osaka, Japan; 13 Department of Emergency Medicine, Fukuchiyama City Hospital, Fukuchiyama, Japan; 14 Division of Environmental Medicine and Population Sciences, Department of Social and Environmental Medicine, Graduate School of Medicine, Osaka University, Osaka, Japan; 15 Department of Healthcare Epidemiology, School of Public Health in the Graduate School of Medicine, Kyoto University, Kyoto, Japan

**Correction to: J Intensive Care**

**https://doi.org/10.1186/s40560-019-0384-2**

In the original publication of this article [1], the figure legends of Fig. 2 and Fig. 3 are wrong and should be changed as below:

**Fig. 2:**
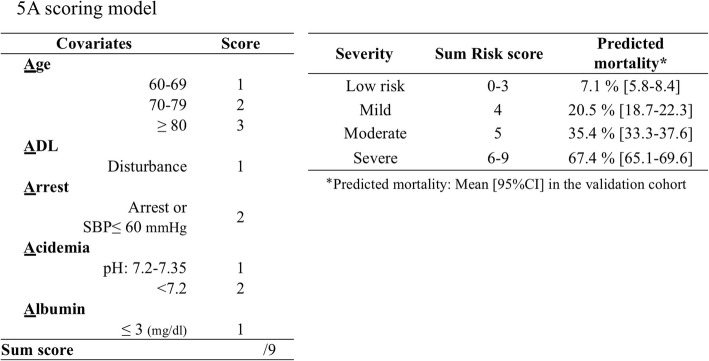
5A scoring model

**Fig. 3:**
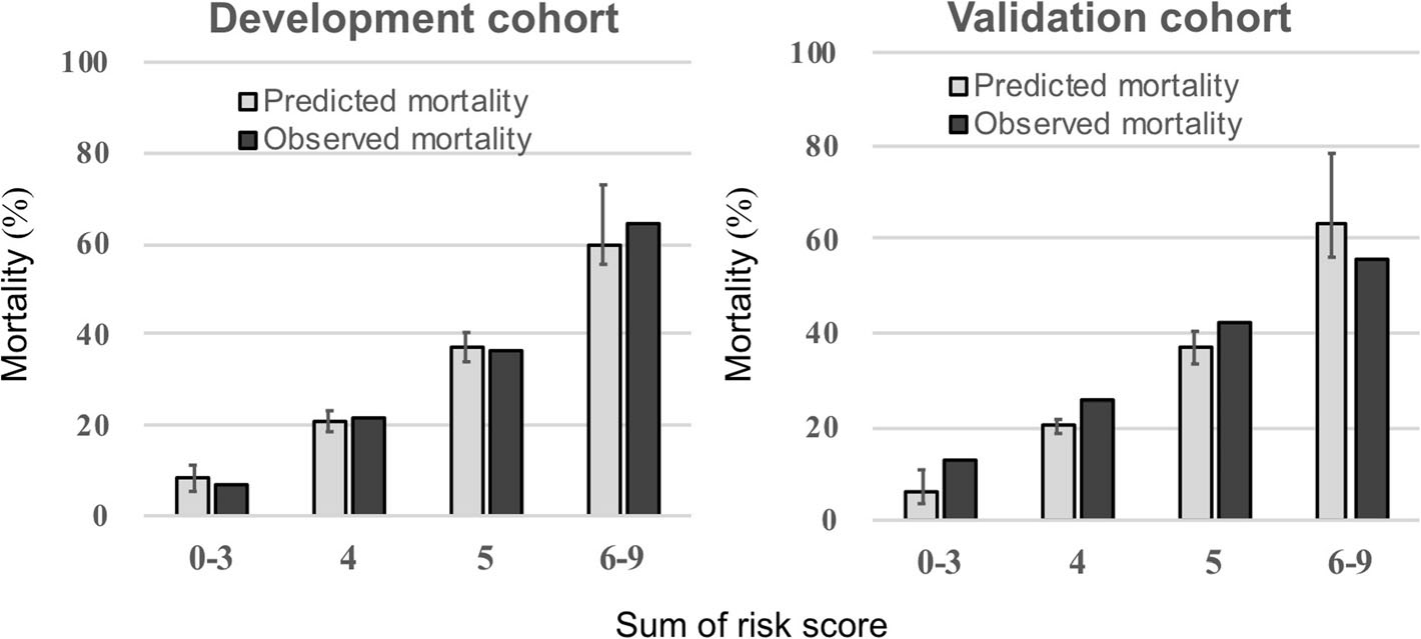
Predicted and observed mortality based on the 5A scoring system
