# Supplementary material for: The development and validation of a “5A” severity scale for predicting in-hospital mortality after accidental hypothermia from J-point registry data
Source: J Intensive Care. 2019 May 3;7:27. doi: 10.1186/s40560-019-0384-2 (PMC6499959; doi:10.1186/s40560-019-0384-2)
Supplement: Supplementary file 1 — The definition of patient characteristics and laboratory data. Table S1. The range of the laboratory data on arrival at the emergency department. Table S2. Coefficient β and adjusted odds ratio with 95% confidence intervals. Table S3. Model performance in the development cohort assessed by bootstrap and that in validation cohort. Figure S1. Calibration Plot in each cohort. Table S4. The conversion of the coefficient values to the score. Table S5. Comparing the discrimination performance in validation cohort. (DOCX 353 kb) [file 40560_2019_384_MOESM1_ESM.docx]

**Additional file**

**The definition of patient characteristics and laboratory data**

Activities of daily living (ADL) was defined as daily activity including eating, dressing, getting into or out of a bed or chair, taking a bath or shower and using toilet independently. Disturbance is assessed as the requirement of partial or total assistance for these activities before the AH event, based on the judgement of the physicians or nurses in charge or their families.

The comorbidities included cardiovascular diseases (ischemic heart disease, heart failure, arrhythmia, hypertension, and other cardiovascular diseases), neurological diseases (stroke, epilepsy, Parkinson disease or syndrome, and other neurological diseases), endocrine diseases (diabetes mellitus, thyroid disease, adrenal insufficiency, and other endocrine diseases), psychiatric diseases (depression, schizophrenia, and other psychiatric diseases), malignant diseases, dementia, and other diseases recorded in the chart.

Japan coma scale (JCS) is a ten-scale tool (similar to the AVPU scale), which uses one digit (0–3) to reflect alertness or eye-opening, two digits (10–30) to reflect eye-opening in response to verbal or pain stimuli, and three digits (100–300) to reflect no response to verbal or pain stimuli. This tool is most commonly used among Japanese paramedics and medical staff, and is well associated with the GCS.

The serum pH was measured by the blood gas sample initially obtained on the arrival at the emergency department. Either atrial or venous blood sample were allowable. It is because pH on the blood gas assessment is known as well correlation between venous and atrial blood sample, and the venous blood sample can be thought as reasonable substitute other than PaO_2_.[1]

**Table S1.** The range of the laboratory data on arrival at the emergency department

| **Parameters** | | **Development cohort** | **Validation cohort** | **Total cohort** |  |
| --- | --- | --- | --- | --- | --- |
|  | Median (2.5-97.5 percentile) | **(N= 288)** | **(N= 244)** | **(N= 532)** |  |
| **pH** | | 7.31 (6.90-7.46) | 7.31 (6.79-7.48) | 7.31 (6.86-7.47) |  |
| **K^+^ (mmol/l)** | | 4.2 (2.3-7.0) | 4.0 (2.5-7.3) | 4.1 (2.5-7.0) |  |
| **Albumin (g/dl)** | | 3.4 (1.7-5.0) | 3.5 (1.7-5.0) | 3.4 (1.7-5.0) |  |

**Formula for predicted in-hospital mortality**

Predicted in-hospital mortality: p= 1/{1+Exp-(ax+b)}

ax+b=- 4.2338

+Age(60-69y.o)*0.675+Age(70-79y.o)*1.376+Age(≥80 y.o)*1.976

+ADL(distrubance)*0.918

+SBP(61-90mmHg)*(-0.146)+near-Arrest*1.716

+ pH(7.2-7.35)*0.786+pH(<7.2)*1.217+pH(Unknown)*(-0.006)

+ Alb(≤3 mg/dl)*0.952+Alb(Unknown)*0.146

ADL:Activity of daily living, SBP: systolic blood pressure

near-Arrest: systolic blood pressure ≤ 60 mmHg, unmeasurable, and cardiac arrest,

Alb: serum Albumin

**Table S2.** Coefficient β and adjusted odds ratio with 95% confidence intervals

| **Covariates** | | **coefficientsβ** | **AOR** |  | **95%CI** | | |  |
| --- | --- | --- | --- | --- | --- | --- | --- | --- |
| **Intercept** | | -4.233 |  |  |  |  |  |  |
| **Age** | |  |  |  |  |  |  |  |
|  | < 60 y.o | (reference) |  |  |  |  |  |  |
|  | 60-69 | 0.675 | 1.964 | [ | 0.355 | - | 10.851 | ] |
|  | 70-79 | 1.376 | 3.958 | [ | 0.961 | - | 16.311 | ] |
|  | ≥ 80 | 1.976 | 7.216 | [ | 1.826 | - | 28.514 | ] |
| **ADL** | |  |  |  |  |  |  |  |
|  | Independent | (reference) |  |  |  |  |  |  |
|  | Disturbance | 0.918 | 2.505 | [ | 1.300 | - | 4.826 | ] |
| **SBP** | |  |  |  |  |  |  |  |
|  | ≥ 90 mmHg | (reference) |  | [ | 1.000 | - | 1.000 | ] |
|  | 61-90 | -0.146 | 0.864 | [ | 0.359 | - | 2.080 | ] |
|  | near Arrest | 1.716 | 5.563 | [ | 2.103 | - | 14.718 | ] |
| **pH** | |  |  |  |  |  |  |  |
|  | > 7.35 | (reference) |  |  |  |  |  |  |
|  | 7.2-7.35 | 0.786 | 2.195 | [ | 1.008 | - | 4.779 | ] |
|  | <7.2 | 1.217 | 3.375 | [ | 1.201 | - | 9.485 | ] |
|  | Unknown | -0.006 | 0.994 | [ | 0.309 | - | 3.200 | ] |
| **Alb** | |  |  |  |  |  |  |  |
|  | > 3 mg/dl | (reference) |  |  |  |  |  |  |
|  | ≤ 3 | 0.952 | 2.590 | [ | 1.278 | - | 5.251 | ] |
|  | Unknown | 0.146 | 1.158 | [ | 0.474 | - | 2.827 | ] |

AOR: adjusted odds ratio, CI: confidence interval, ADL: activities of daily living,

SBP: systolic blood pressure, near arrest: SBP of ≤60 mmHg, unmeasurable values, and confirmed arrest. Alb: albumin. Akaike’s Information Criterion (AIC): –534.9

**Table S3.** Model performance in the development cohort assessed by bootstrap and that in validation cohort

| **Cohort** | Development | | | Validation |
| --- | --- | --- | --- | --- |
| **Index** | Original | Optimism | Bias Corrected index | Original |
| **Dxy** | 0.583 | 0.091 | 0.493 | 0.482 |
| **C index** | 0.792 | 0.046 | 0.746 | 0.741 |
| **R^2^** | 0.268 | 0.095 | 0.173 | 0.145 |
| **Intercept** | 0 | 0.23 | -0.23 | -0.15 |
| **Slope** | 1 | 0.237 | 0.763 | 0.646 |
| **Brier score** | 0.139 | -0.015 | 0.155 | 0.172 |

Model performance was assessed by bootstrap 200 repetitions in development cohort as an internal validation.

**Fig S1.** Calibration Plot in each cohort

**
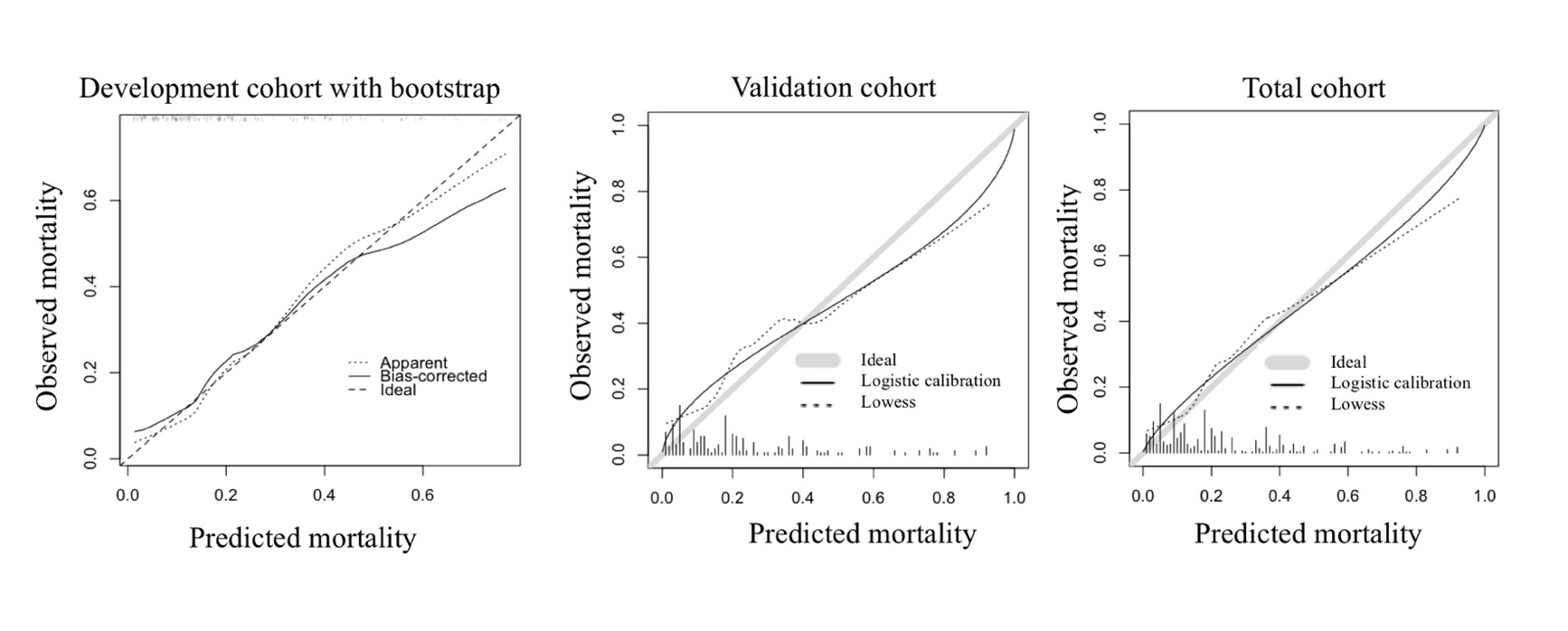

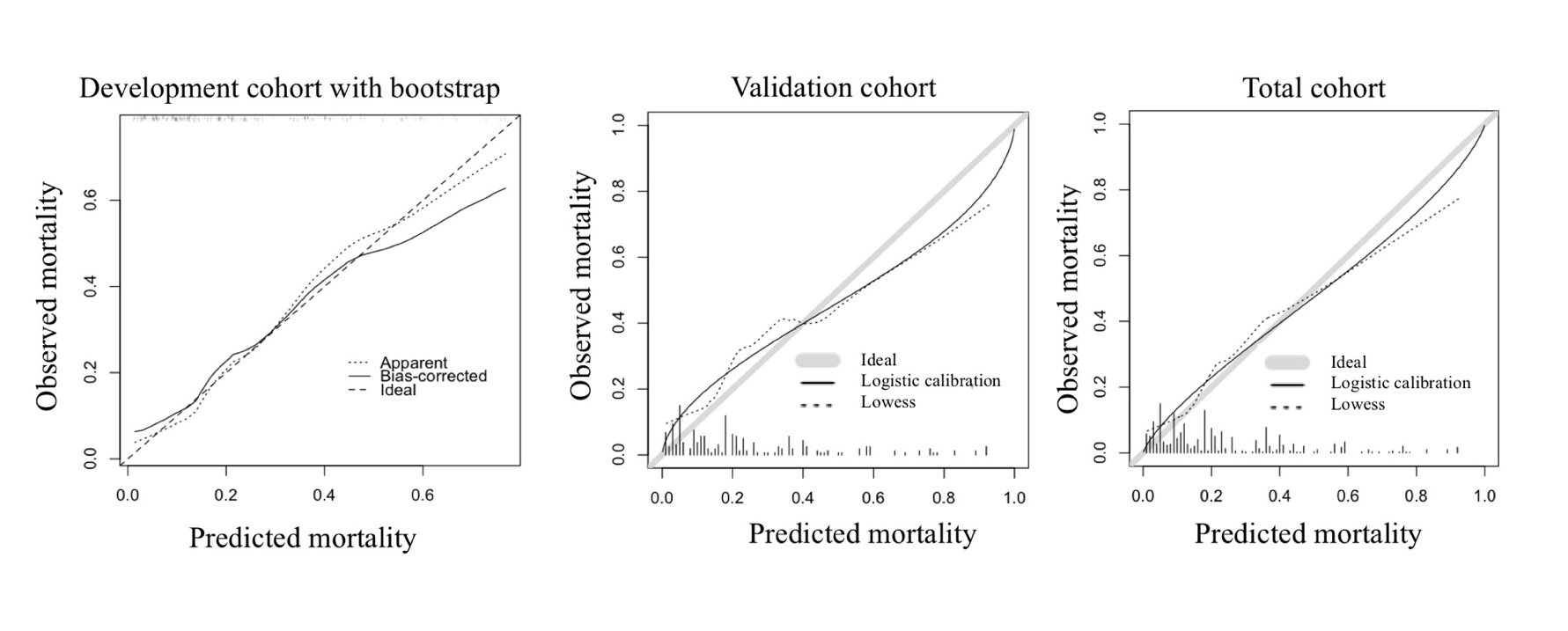
**

In the development cohort, the ideal dashed line reflects perfect calibration between the predicted and observed mortality rates. The apparent performance, indicated by the dotted line, reflects the calibrated performance of the model. The solid line reflects the bias-corrected performance based on bootstrapping. The validation cohort also has ideal dashed lines. The solid lines reflect the fitted logistic calibration curve. The dotted lines reflect a smooth nonparametric fit using a locally weighted scatter plot for smoothing.

**Table S4.** The conversion of the coefficient values to the score

| Beta-coefficient value | Score |
| --- | --- |
| <0.35 | 0 |
| 0.7±0.35 (0.35-1.05) | 1 |
| 1.4±0.35 (1.05-1.75) | 2 |
| 2.1±0.35 (1.75-2.45) | 3 |

We converted the coefficient values to the score by rounding off (Coefficient value/0.7) to the nearest integer number to make simple scoring system.

**Table S5.** Comparing the discrimination performance in validation cohort

| Model | c-statistics | 95%CI | The difference* | 95%CI* |
| --- | --- | --- | --- | --- |
| 5A scoring | 0.731 | (0.655-0.795) | reference | - |
| Swiss staging* | 0.558 | (0.479-0.635) | 0.172 | (0.078-0.266) |

Core body temperature graded by Swiss staging system (32-35℃, 28-32, 24-28, <24)[2]

CI: confidence interval

*Comparisons between the models was evaluated with Pearson’s chi squared test.

<Supplementary Reference>

1. Cowley NJ, Owen A, Bion JF: **Interpreting arterial blood gas results**. *BMJ : British Medical Journal* 2013, **346**:f16.

2. Brown DJ, Brugger H, Boyd J, Paal P: **Accidental hypothermia**. *N Engl J Med* 2012, **367**(20):1930-1938.
